# A Simulation Approach to Evaluate Pre-Enrollment Survival Bias in Cross-National Aging Epidemiology Research

**DOI:** 10.1093/geroni/igaf122.540

**Published:** 2025-12-31

**Authors:** Ryan Andrews, Kelvin Zhang, Alden Gross, Sneha Mani, Jennifer Weuve, Lindsay Kobayashi

**Affiliations:** Columbia University, New York, New York, United States; University of Michigan, Ann Arbor, Michigan, United States; Johns Hopkins Bloomberg School of Public Health, Baltimore, Maryland, United States; Johns Hopkins Bloomberg School of Public Health, Baltimore, Maryland, United States; Boston University, Boston, Massachusetts, United States; University of Michigan, Ann Arbor, Michigan, United States

## Abstract

Selection bias caused by collider stratification can distort the estimated effects of exposures on outcomes. As a specific type of selection bias, pre-enrollment survival bias is especially salient in aging research because differential mortality in older populations can yield study samples that systematically deviate from their target populations. Several cross-national comparison studies have observed heterogeneity in the estimated effect of risk factors on aging outcomes, which could be due to true effect heterogeneity or spurious differences due to bias. For example, inconsistent estimates across countries could arise from various magnitudes of pre-enrollment survival bias induced by varying life expectancies across countries. To evaluate how pre-enrollment survival bias can account for the discrepancy in effect estimation in multiple countries, we used cross-national simulations to quantify potential bias in the estimated effect of type 2 diabetes duration on dementia. We simulated country-specific cohorts of individuals born between 1927 and 1951 in the US, England, and India (N = 250,000 each). Under three causal structures for each country that represented the relations among type 2 diabetes duration, vital status in 2016, and dementia status in 2016, we compared the estimated effect among survivors with the true causal effect. We found that the effect of type 2 diabetes duration on dementia could be underestimated as much as 4.5 times the estimated standard error. Age standardization was also applied to enable comparisons of estimated effects across countries. The age-standardized estimates in the US and England did not differ substantially.

